# Exposure to Polybrominated Diphenyl Ethers and Risk of All-Cause and Cause-Specific Mortality

**DOI:** 10.1001/jamanetworkopen.2024.3127

**Published:** 2024-04-01

**Authors:** Buyun Liu, Hans-Joachim Lehmler, Ziyi Ye, Xing Yuan, Yuxiang Yan, Yuntian Ruan, Yi Wang, Yu Yang, Shuhan Chen, Wei Bao

**Affiliations:** 1 Department of Nursing, The First Affiliated Hospital of USTC, Institute of Public Health Sciences, Division of Life Sciences and Medicine, University of Science and Technology of China, Hefei, Anhui, China; 2 Department of Occupational and Environmental Health, College of Public Health, University of Iowa, Iowa City; 3 Institute of Public Health Sciences, Division of Life Sciences and Medicine, University of Science and Technology of China, Hefei, Anhui, China; 4 Department of Endocrinology, The First Affiliated Hospital of USTC, Institute of Public Health Sciences, Division of Life Sciences and Medicine, University of Science and Technology of China, Hefei, Anhui, China

## Abstract

**Importance:**

Polybrominated diphenyl ethers (PBDEs) are an important group of persistent organic pollutants with endocrine-disrupting properties. However, prospective cohort studies regarding the association of PBDE exposure with long-term health outcomes, particularly mortality, are lacking.

**Objective:**

To examine the association of environmental exposure to PBDEs with risk of all-cause and cause-specific mortality.

**Design, Setting, and Participants:**

This nationally representative cohort study used data from the National Health and Nutrition Examination Survey 2003 to 2004 and linked mortality information through December 31, 2019. Adults aged 20 years or older with available data on PBDE measurements and mortality were included. Statistical analysis was performed from February 2022 to April 2023.

**Exposures:**

PBDE analytes in serum samples were measured using solid phase extraction and isotope dilution gas chromatography high-resolution mass spectrometry.

**Main Outcomes and Measures:**

All-cause mortality, cancer mortality, and cardiovascular mortality.

**Results:**

This study included 1100 participants (mean [SE] age, 42.9 [0.6] years; proportion [SE] female, 51.8% [1.6%]; proportion [SE] Hispanic, 12.9% [2.7%]; proportion [SE] non-Hispanic Black, 10.5% [1.6%]; proportion [SE] non-Hispanic White, 70.8% [3.7%]; proportion [SE] other race and ethnicity, 5.8% [1.1%]). During 16 162 person-years of follow-up (median [IQR] follow-up, 15.8 [15.2-16.3] years; maximum follow-up, 17 years), 199 deaths occurred. Participants with higher serum PBDE levels were at higher risk for death. After adjustment for age, sex, and race and ethnicity, lifestyle and socioeconomic factors, and body mass index, participants with the highest tertile of serum PBDE levels had an approximately 300% increased risk of cancer mortality (HR, 4.09 [95% CI, 1.71-9.79]) compared with those with the lowest tertile of serum PBDE levels. No significant association of PBDE exposure with all-cause mortality (HR, 1.43 [95% CI, 0.98-2.07]) or cardiovascular mortality (HR, 0.92 [95% CI, 0.41-2.08]) was observed.

**Conclusions and Relevance:**

In this nationally representative cohort study, PBDE exposure was significantly associated with an increased risk of cancer mortality. Further studies are needed to replicate the findings and determine the underlying mechanisms.

## Key Points

**Question:**

Is environmental exposure to polybrominated diphenyl ether (PBDE) flame retardants associated with risk of all-cause and cause-specific mortality?

**Findings:**

In this cohort study of 16 162 adults in the general US population, higher serum PBDE exposure levels were associated with increased risk of death from cancer during the follow-up.

**Meaning:**

These results suggest evidence for the long-term adverse health effects of PBDEs in humans.

## Teaser

This cohort study uses data from the National Health and Nutrition Examination Survey (NHANES) to investigate the association of environmental exposure to polybrominated diphenyl ether (PBDE) flame retardants with risk of all-cause and cause-specific mortality.

## Introduction

Polybrominated diphenyl ethers (PBDEs) are a group of persistent organic pollutants that represent a substantial environmental and human health concern.^1,2^ Since the 1970s, PBDEs have been used as flame retardants in a wide array of consumer products, such as building materials, furnishings, and electronics.^3^ PBDEs enter the environment at sites of production, use, and disposal of PBDE-containing products. PBDEs strongly bind to sediments and soils because of their low water solubility and high lipophilicity.^4^ They degrade in the environment by debromination reactions, forming more toxic, lower brominated PBDE congeners.

Although the manufacturing and use of PBDEs are mostly banned under the Stockholm Convention on Persistent Organic Pollutants, PBDEs remain ubiquitous in the environment (eg, air, water, soil), bioaccumulate in food chains, and have a high potential for long-range transport.^5^ Humans continue to be exposed to these compounds from eating foods or breathing air contaminated with PBDEs.^3,6,7,8^ Biomonitoring studies among a nationally representative sample from the National Health and Nutrition Examination Surveys (NHANES) have shown that several PDBE congeners (ie, BDE28, BDE47, BDE99, BDE100, BDE153) are frequently detected in the US general population.^9,10^

The health effects of PBDE exposure in humans remain unclear. Several cross-sectional studies found that PBDE exposure was associated with increased risk of thyroid disease,^11^ diabetes,^12,13^ and metabolic syndrome.^12^ In several case-control studies for the association between PBDE exposure and cancer risk, some but not all reported a significant association of PBDE exposure with thyroid and breast cancer.^14,15,16,17,18^ However, prospective cohort studies investigating the association between PBDE exposure and long-term risk of mortality are lacking. In this study, we investigated the association of PBDE exposure, assessed by serum PBDE levels, with all-cause and cause-specific mortality in a nationally representative cohort of US adults.

## Methods

### Study Population

The NHANES is a nationally representative health survey of the civilian noninstitutionalized resident population in the US, administered by the National Center for Health Statistics (NCHS) at the Centers for Disease Control and Prevention (CDC). The uniqueness of the NHANES is that it not only collects questionnaire data through in-person interviews but also performs health examinations in the Mobile Examination Center and collects specimens for laboratory tests. The NCHS ethics review board has approved the NHANES protocol. Written informed consent was obtained from all participants. This study was determined to be exempt because the data were deidentified. We followed the Strengthening the Reporting of Observational Studies in Epidemiology (STROBE) reporting guideline.

For this analysis, we included adults aged 20 years and older who participated in the NHANES 2003 to 2004 and had available data on PBDE measurements. We linked all participants to mortality data through 2019, which allowed approximately 16 years of observation for mortality outcomes. Individuals with cardiovascular disease or cancer at baseline were excluded. A flow diagram about participant inclusion has been provided in the eFigure in Supplement 1.

### Assessment of PBDE Exposure

PBDE analytes in serum samples were measured by high-resolution gas chromatography–high-resolution mass spectrometry using isotope dilution for quantification. Details of the laboratory methods of PBDE measurement in NHANES were published on the official website. The lower limit of detection (LLOD) for PBDEs was 0.36 μg/L in 2003 to 2004. For PBDE analytes below the LLOD, NHANES assigned a value of the LLOD divided by the square root of 2. PBDEs were reported on a lipid-adjusted basis using concentrations of serum total cholesterol and triglycerides.

PBDE levels were the sum of the 10 PBDEs (2,2’,4-tribromodiphenyl ether; 2,4,4’-tribromodiphenyl ether; 2,2’,4,4’-tetrabromodiphenyl ether; 2,2’,3,4,4’-pentabromodiphenyl ether; 2,2’,4,4’,5-pentabromodiphenyl ether; 2,2’,4,4’,6-pentabromodiphenyl ether; 2,2’,4,4’,5,5′-hexabromodiphenyl ether; 2,2’,4,4’,5,6’-hexabromodiphenyl ether; 2,2’,3,4,4’,5′,6-heptabromodiphenyl ether; 2,3′,4,4’-tetrabromodiphenyl ether) measured in the NHANES 2003-2004. Five PBDEs for which at least 60% of the study participants had concentrations more than the limit of detection were analyzed individually.

### Ascertainment of Mortality Outcomes

We used the NHANES Public-Use Linked Mortality File through December 31, 2019, which was linked by the NCHS to the National Death Index with a probabilistic matching algorithm to determine the mortality status.^19^ National Death Index is an NCHS centralized database of all deaths in the US. Data about underlying cause of death were used for case definition according to the *International Statistical Classification of Diseases and Related Health Problems, Tenth Revision (ICD-10)*.^20^ Accordingly, the NCHS classified cardiovascular mortality as death from heart disease (*ICD-10* codes I00-I09, I11, I13, I20-I51) or cerebrovascular disease (*ICD-10* codes I60-I69) and cancer mortality as death from malignant neoplasms (*ICD-10* codes C00-C97). This approach has been used in previous reports.^21,22^

### Assessment of Covariates

Information on age, sex, race and ethnicity, education, family income, smoking status, alcohol drinking, physical activity, and dietary intake was self-reported using questionnaires. According to the 1997 US federal Office of Management and Budget standards, race and ethnicity was categorized into Hispanic (including Mexican and non-Mexican Hispanic), non-Hispanic Black, non-Hispanic White, and other. Information about race and ethnicity was assessed considering the differences in mortality rates among different ethnic groups. Family income was categorized as the ratio of family income to federal poverty level less than 1.0, 1.0 to 1.9, 2.0 to 3.9, and greater than or equal to 4.0. A higher income-to-poverty ratio indicates a better family income status. Self-reported education status was grouped as lower than high school, high school, and college or higher. In accordance with the NCHS classifications, individuals who smoked less than 100 cigarettes in their lifetime were defined as having never smoked; those who had smoked more than 100 cigarettes, but did not smoke at the time of survey, were considered as having formerly smoked; and those who had smoked 100 cigarettes in their lifetime and smoked cigarettes at the time of survey were considered currently smoking. Alcohol intake was categorized as none (0 g/d), moderate drinking (0.1 to 27.9 g/d for men and 0.1 to 13.9 g/d for women), and heavy drinking (≥28 g/d for men and ≥14 g/d for women). For physical activity, participants were asked an array of questions related to daily activities in the questionnaire. Physical activity for each participant was categorized as follows: (1) below, 150 minutes per week moderate- to vigorous-intensity activity; (2) meet, 150 to 300 minutes per week moderate- to vigorous-intensity activity; or (3) exceed, 300 minutes per week moderate- to vigorous-intensity activity. Dietary information was collected by 24-hour dietary recall interviews, from which total energy intake was calculated using the US Department of Agriculture Automated Multiple-Pass Method. We used the Healthy Eating Index-2010 (HEI-2010) to indicate the overall quality of diet (HEI-2010 score from 0 to 100, with 100 being the best-quality diet).^23^ Body weight and height were measured by trained health technicians following the NHANES Anthropometry Procedures Manual. Body mass index (BMI) was calculated as weight in kilograms divided by the square of height in meters.

### Statistical Analysis

NHANES used a complex, multistage probability sampling design to represent the national, civilian, noninstitutionalized population in the US. Therefore, sample weights, strata, and primary sampling units were applied following the NHANES Analytic Guidelines^24^ to account for the unequal probability of selection, oversampling of certain subpopulations, and nonresponse adjustment.

We created a summary measure of total PBDE concentrations (ng/g) by summing the concentrations for all the 10 PBDE analytes. Additionally, we analyzed the association between 5 PBDE congeners and mortality, respectively, because only these 5 congeners had a detection rate greater than 60%: 2,4,4’-tribromodiphenyl ether (PBDE-28); 2,2′,4,4′-tetrabromodiphenyl ether (PBDE-47); 2,2',4,4',5-pentabromphenyl (BR99); 2,2′,4,4′,6-pentabromodiphenyl ether (PBDE-100); and 2,2’,4,4’,5,5′-hexabromphenyl (PBDE-153).

Means and proportions of baseline characteristics were compared by using linear regression for continuous variables and logistic regression for categorical variables. We used Cox proportional hazards regression models to estimate hazard ratios (HRs) and 95% CIs for the associations between PBDE exposure and risk of mortality. Follow-up time for each person was calculated as the difference between the NHANES examination date and the last known date alive or censored from the linked mortality file. The base model was adjusted for age, sex, and race and ethnicity. The second model was additionally adjusted for education, family income level, smoking status, alcohol intake, physical activity, total energy intake, and overall diet quality indicated by HEI-2010 score. BMI was entered into the model separately because it can also be an intermediate on the pathway between PBDE exposure and mortality risk. To test linear trends across categories of PBDE concentrations, we assigned the median values for each category and fitted the log-transformed median values as continuous variable in models. Furthermore, we performed stratified analyses and interaction analyses to examine whether the association differed by age, sex, race and ethnicity, diet quality, physical activity, and obesity status. We conducted a sensitivity analysis by excluding participants younger than 40 years, considering they were less likely to die even after 16 years of follow-up. All statistical analyses were conducted using survey modules of SAS software version 9.4 (SAS Institute) from February 2022 to April 2023. A 2-sided *P* < .05 was considered statistically significant.

## Results

This study included 1100 adults (mean [SE] age, 42.9 [0.6] years; proportion [SE] female, 51.8% [1.6%]; proportion [SE] Hispanic, 12.9% [2.7%]; proportion [SE] non-Hispanic Black, 10.5% [1.6%]; proportion [SE] non-Hispanic White, 70.8% [3.7%]; proportion [SE] other race and ethnicity, 5.8% [1.1%]). During 16 162 person-years of follow-up (median [IQR] follow-up, 15.8 [15.2-16.3] years; maximum follow-up, 17 years), 199 deaths occurred, including 64 deaths from cardiovascular disease (CVD) and 52 deaths from cancer. Participants with higher serum PBDE levels were more likely to be younger, male, and have poorer dietary quality (Table 1). Participant characteristics by PBDE congeners are shown in eTable 1 in Supplement 1.

**Table 1.** Characteristics of the Study Population

Characteristic	No. of participants	Total PBDE, % (SE)			*P* value
		Tertile 1	Tertile 2	Tertile 3	
No. of participants	1100	367	366	367	
Age, mean (SE), y	1100	45.1 (0.9)	42.1 (0.8)	41.2 (1.1)	.004
Sex					
Male	516	48.3 (3.0)	43.3 (3.3)	53.0 (2.5)	.09
Female	584	51.7 (3.0)	56.7 (3.3)	47.0 (2.5)	
Race and ethnicity					
Hispanic	293	10.9 (2.6)	14.3 (3.2)	13.5 (3.2)	.36
Non-Hispanic Black	210	8.9 (2.3)	10.5 (1.6)	12.2 (2.2)	
Non-Hispanic White	544	74.9 (4.3)	67.9 (4.2)	69.5 (4.5)	
Other^a^	53	5.3 (1.6)	7.3 (2.1)	4.7 (1.3)	
Education					
Less than high school	305	14.0 (2.1)	18.1 (1.4)	16.7 (2.8)	.37
High school	275	29.4 (3.4)	22.7 (3.8)	28.2 (3.3)	
College or higher	520	56.6 (2.9)	59.1 (4.3)	55.1 (3.1)	
Family income to poverty ratio					
<1.0	207	11.9 (2.1)	13.9 (2.3)	14.2 (2.0)	.49
1.0-1.9	260	16.8 (1.8)	17.8 (2.6)	19.9 (2.2)	
2.0-3.9	281	27.8 (3.0)	31.9 (3.9)	26.9 (1.9)	
≥4.0	286	36.4 (3.3)	32.4 (4.8)	32.5 (2.4)	
Missing	66	7.1 (1.5)	3.9 (1.8)	6.6 (1.6)	
Smoking					
Never	580	50.9 (4.2)	49.8 (2.9)	52.3 (2.9)	.52
Ever	243	23.5 (3.7)	22.4 (3.1)	17.7 (2.5)	
Current	277	25.6 (3.4)	27.8 (3.1)	30.0 (2.5)	
Alcohol drinking					
No	790	67.6 (4.6)	71.1 (4.0)	69.3 (3.1)	.63
Moderate	97	9.9 (1.7)	8.9 (2.2)	10.1 (1.7)	
Heavy	169	20.2 (3.5)	16.1 (2.9)	18.9 (3.1)	
Missing	44	2.3 (0.9)	3.9 (1.1)	1.6 (0.7)	
Physical activity categories^b^					
Below	518	40.8 (3.6)	38.0 (3.1)	44.1 (3.0)	.36
Meet	174	18.1 (3.1)	18.9 (2.3)	14.2 (2.1)	
Exceed	408	41.1 (4.1)	43.1 (2.6)	41.7 (3.5)	
Total energy intake, kcal/d	1100	2381.8 (78.0)	2230.3 (83.4)	2385.1 (43.2)	.15
HEI-2010 score	1100	46.6 (1.0)	44.3 (1.2)	45.2 (0.8)	.048
BMI categories					
<25	349	34.1 (2.6)	34.0 (2.0)	32.9 (3.5)	.99
25-29.9	375	32.6 (2.7)	31.4 (3.2)	32.5 (3.2)	
≥30	356	31.8 (3.9)	33.3 (3.4)	33.4 (3.2)	
Missing	20	1.4 (0.8)	1.3 (0.7)	1.2 (0.4)	

Abbreviations: BMI, body mass index (calculated as weight in kilograms divided by height in meters squared); HEI-2010, healthy eating index 2010; MET, metabolic equivalent of task.

^a^ Values were weighted mean (SE) for continuous variables and weighted percentages (SE) for categorical variables, except the number of participants.

^b^ Other race and ethnicity included non-Hispanic respondents who were self-identified with more than 1 race.

^c^ Physical activity for each participant was categorized as follows: (1) below, 150-min/wk moderate-intensity to vigorous-intensity activity; (2) meet, 150-300-min/wk moderate-intensity to vigorous-intensity activity; or (3) exceed, 300-min/wk moderate-intensity to vigorous-intensity activity.

The association between PBDE exposure and all-cause and cause-specific mortality is shown in Table 2. Participants with higher serum PBDE levels were at a higher risk for death from cancer during the follow-up. After adjustment for age, sex, race and ethnicity, socioeconomic status, dietary and lifestyle factors, and BMI, the multivariable-adjusted HR of cancer mortality comparing the highest tertile with the lowest tertile of serum PBDE levels was 4.09 (95% CI, 1.71 to 9.79). No significant association was observed for all-cause mortality (multivariable-adjusted HR, 1.43 [95% CI, 0.98-2.07]) or CVD mortality (multivariable-adjusted HR, 0.92 [95% CI, 0.41-2.08]). Stratified analyses showed that the observed associations between PBDE exposure and cancer mortality did not differ appreciably by age, sex, race and ethnicity, diet quality, physical activity, or obesity status (Table 3). Stratified analyses for all-cause mortality and CVD mortality are shown in eTables 2 and 3 in Supplement 1, and no association of PBDE exposure with all-cause mortality and CVD mortality in any stratum was found.

**Table 2.** Association of Serum PBDE Levels With All-Cause and Cause-Specific Mortality^a^

	Risk of mortality, HR (95% CI)			HR per unit increase	*P* value for trend
	Tertile 1	Tertile 2	Tertile 3		
PBDE level, median (range), ng/mL	16.7 (5.6-24.9)	37.8 (25.0-59.6)	103.5 (≥59.7)
**All-cause mortality**					
Deaths/person-years	74/5296	55/5512	70/5353	NA	NA
Model 1	1 [Reference]	0.89 (0.59-1.34)	1.35 (0.91-1.98)	1.18 (0.94-1.48)	.14
Model 2	1 [Reference]	0.86 (0.57-1.29)	1.41 (0.96-2.07)	1.21 (0.96-1.52)	.10
Model 3	1 [Reference]	0.86 (0.59-1.26)	1.43 (0.98-2.07)	1.22 (0.97-1.53)	.08
**CVD mortality**					
Deaths/person-years	25/5296	21/5512	18/5353	NA	NA
Model 1	1 [Reference]	0.83 (0.29-2.39)	0.84 (0.31-2.24)	0.90 (0.51-1.60)	.90
Model 2	1 [Reference]	0.73 (0.25-2.12)	0.95 (0.40-2.27)	0.94 (0.55-1.63)	.95
Model 3	1 [Reference]	0.74 (0.28-2.00)	0.92 (0.41-2.08)	0.93 (0.57-1.54)	.93
**Cancer mortality**					
Deaths/person-years	12/5296	16/5512	24/5353	NA	NA
Model 1	1 [Reference]	2.00 (1.00-3.98)	3.26 (1.59-6.70)	1.86 (1.29-2.69)	.003
Model 2	1 [Reference]	1.95 (0.89-4.29)	4.02 (1.59-10.18)	2.13 (1.25-3.63)	.009
Model 3	1 [Reference]	1.97 (0.91-4.28)	4.09 (1.71-9.79)	2.15 (1.29-2.56)	.006

Abbreviations: CVD, cardiovascular disease; HR, hazard ratio; NA, not applicable.

^a^ Model 1 was adjusted for age, sex, and race and ethnicity; model 2: model 1 plus education, family income status, smoking, alcohol drinking, physical activity, total energy intake, and healthy eating index 2010 score; model 3: model 2 plus body mass index.

**Table 3.** Stratified Analyses for the Association of Serum PBDE Levels With Cancer Mortality^a^

Characteristic	Tertile 1		Tertile 2		Tertile 3		*P* value for interaction
	Deaths/person-years	HR (95% CI)	Deaths/person-years	HR (95% CI)	Deaths/person-years	HR (95% CI)	
Age, y							
<60	4/3961^b^	1 [Reference]	6/4531	0.96 (0.29-3.18)	6/4335	1.22 (0.14-10.41)	.35
≥60	8/1336	1 [Reference]	10/981	3.10 (0.93-10.34)	18/1019	8.53 (1.83-39.80)	
Sex							
Male	6/2380	1 [Reference]	11/2371	5.97 (1.81-19.67)	15/2717	6.82 (2.72-17.11)	.89
Female	6/2916	1 [Reference]	5/3141	1.41 (0.20-10.15)	9/2637	4.85 (1.22-19.30)	
Race and ethnicity							
White	4/2842	1 [Reference]	6/2522	2.54 (0.49-13.14)	11/2587	4.60 (1.25-16.88)	.62
Other race and ethnicity	8/2454	1 [Reference]	10/2990	0.85 (0.18-3.91)	13/2767	1.32 (0.20-8.88)	
Diet quality^c^							
Lower	4/2641	1 [Reference]	9/3219	1.94 (0.44-8.52)	12/2824	4.58 (1.28-16.35)	.92
Higher	8/2656	1 [Reference]	7/2293	4.30 (0.81-22.76)	12/2530	6.94 (2.07-23.29)	
Physical activity^d^							
Lower	5/3378	1 [Reference]	11/3346	5.76 (1.37-24.30)	15/3332	10.42 (2.61-41.55)	.10
Higher	7/1918	1 [Reference]	5/2166	0.54 (0.10-2.94)	9/2021	1.13 (0.34-3.77)	
Obesity							
BMI <30	9/3546	1 [Reference]	11/3470	2.43 (0.85-6.99)	18/3549	3.90 (1.29-11.78)	.64
BMI ≥30	3/1686	1 [Reference]	5/1921	7.79 (0.63-97.69)	5/1723	21.88 (1.88-255.4)	

Abbreviations: BMI, body mass index (calculated as weight in kilograms divided by height in meters squared); PBDE, polybrominated diphenyl ether.

^a^ Adjusted for age, sex, race/ethnicity, education, family income status, smoking, alcohol drinking, physical activity, total energy intake, healthy eating index score, and BMI.

^b^ The number of events (deaths) per number of person-years at risk in each tertile of exposure.

^c^ Lower or higher diet quality was defined as the healthy eating index score less than the median score or greater than or equal to the median score, respectively.

^d^ Lower or higher physical activity level was defined as below or meeting the physical activity guidelines, respectively.

Individual congener analyses showed that higher 2,2’,4,4’-tetrabromophenyl ether (PBDE 47) exposure was associated with an increased risk of cancer mortality (HR, 3.21 [95% CI, 1.29-7.96]); and 2,2’,4,4’,6-pentabromophenyl (PBDE 100) was associated with all-cause mortality (HR, 1.60 [95% CI, 1.10-2.31]) and cancer mortality (HR, 3.81 [95% CI, 1.16-12.49]), comparing the highest tertile with the lowest tertile levels. Similarly, 2,2’,4,4’,5,5′-hexabromophenyl (PBDE 153) was associated with all-cause mortality (HR, 1.78 [95% CI, 1.16-2.73) and cancer mortality (HR, 4.12 [95% CI, 1.37-12.42]). The results are shown in the Figure and details are provided in (eTable 4 in Supplement 1). Sensitivity analyses restricting to individuals aged 40 years or older were consistent with the main analyses (eTable 5 in Supplement 1).

**Figure.**
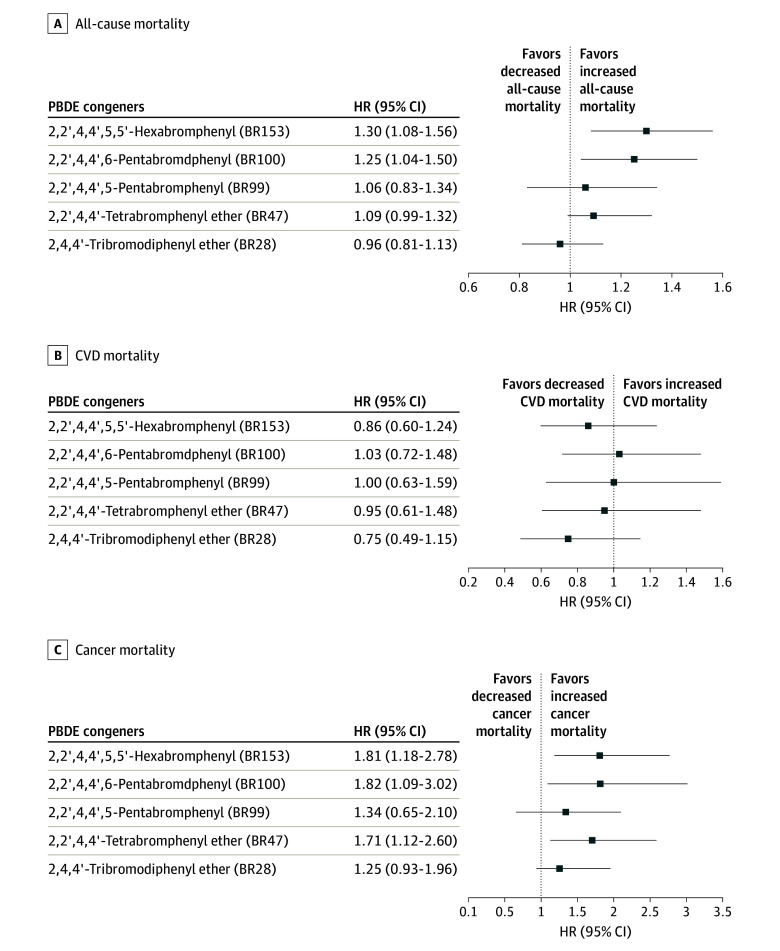
Association of Serum PBDE Congeners Levels With All-Cause and Cause-Specific Mortality CVD indicates cardiovascular disease; HR, hazard ratio; PBDE, polybrominated diphenyl ether.

## Discussion

In this cohort study of a nationally representative sample, we found that PBDE exposure was significantly and positively associated with cancer mortality in adults. The association persisted after adjustment for demographics, socioeconomic status, dietary and lifestyle factors, and BMI. There was no significant association between PBDE exposure and all-cause mortality and CVD mortality.

To our knowledge, this is the first study examining the association of PBDE exposure with risk of cause-specific mortality in the general adult population from the US. One earlier study investigated the association between PBDE exposure and all-cause mortality. However, this study was conducted only among adults aged 60 years or older and did not include cancer and CVD mortality.^25^ Our findings about the positive association between PBDE exposure and cancer mortality align with previous studies showing that PBDE exposure was associated with an increased risk of cancer (eg, thyroid and breast cancer).^18,26,27,28^ However, some studies also reported that PBDE exposure was not associated with cancer risk.^14,29^ The inconsistent findings may be partly related to the study design. Due to the case-control study design, temporal relationship between PBDE exposure and cancer was unclear in most of those previous studies. In this scenario, PBDE levels were measured among participants after cancer diagnosis and treatment, which may not reflect PBDE exposure before the onset of cancer. In addition, cancer type and PBDE congeners that were studied may also contribute to the variations in the findings. In this study, we did not find an association between PBDE exposure and all-cause mortality, which may warrant confirmation in future research with larger sample size. However, individual congener analyses found that PBDE 100 and PBDE 153 were associated with all-cause mortality, which was consistent with findings from the only existing study that was conducted among adults aged 60 years or older.^25^

In vivo and in vitro studies support biological plausibility showing that PBDE exposure could promote cancer occurrence and progression.^30^ As endocrine-disrupting chemicals, PBDEs and their metabolites can bind to hormone receptors (ie, estrogen receptor), act as both agonists and antagonists, and then disrupt hormone homeostasis.^1,2,31^ This plays a role in the development and progression of endocrine tumors such as thyroid cancer.^31^ In addition, PBDEs were found to disrupt facets of genomic integrity and innate immunity in mammary tissue related to breast cancer,^32^ and be carcinogenic in human thyroid cells.^33^ A recent animal study found that the dosage of PBDE-47 was positively associated with tumor sizes,^34^ suggesting its effects on cancer development. Furthermore, a growing number of studies demonstrate that PBDEs could cause oxidative stress, DNA damage, and cell cycle dysregulation.^1,35,36,37,38^ All of these factors play a role in the development and progression of cancer.^39,40^

Our findings have major public health implications. Although PBDEs are mostly banned today under the Stockholm Convention on Persistent Organic Pollutants, their production and use are still ongoing in some regions.^5^ For example, only 13 states in the US have applied limitations on using PBDEs in certain goods, but no federal restrictions are in place.^5^ Despite great concerns, environmental authorities, such as the International Agency for Research on Cancer, the US Department of Health and Human Services, and the US Environmental Protection Agency, stated that there are difficulties in classifying PBDEs as human carcinogens due to the inadequate evidence of carcinogenicity in humans. The present study addresses this knowledge gap by finding an association between PBDEs and cancer mortality in adults from the general US population.

This study has several strengths. We used nationally representative data from the NHANES, which allows us to generalize our findings to a broader population. In addition, the abundant data from the NHANES, including comprehensive information about demographic, socioeconomic, anthropometric measures, and diet and lifestyle factors, provide the opportunity to adjust for various potential confounding factors.

### Limitations

There are also limitations in this study. First, we could not determine the risk of mortality from specific cancer subtypes. Previous studies have reported a significant association between PBDE exposure and increased risk of thyroid and breast cancer.^18,26,27,28^ Therefore, additional studies are warranted to determine the associated risk of cancer mortality by subtypes. Second, the NHANES Linked Mortality File identified causes of death through linkage to the National Death Index, which is based on death certificates. Although this approach has been previously validated by the CDC and used in many CDC reports^41,42,43^ or other relevant literature, we cannot rule out the possibility of errors in classifying the cause of death. Third, cancer mortality in this study represents a composite outcome of death from various cancer types, because the publicly released mortality file provided by the CDC’s National Center for Health Statistics does not include information about death from specific cancer types. Survival rates vary substantially across types of cancer, therefore, future studies are warranted to determine the association of PBDE exposure with mortality from specific types of cancer. Additionally, although many potential confounders were adjusted for, there might still be residual confounding by unmeasured factors.

## Conclusions

In this nationally representative cohort, PBDE exposure was significantly associated with an increased risk of cancer mortality. Further studies are needed to replicate the findings and determine the underlying mechanisms.

## Notes

Supplement 1. **eFigure.** Flow Chart **eTable 1.** Characteristics of the Study Population (n=1,100) **eTable 2.** Stratified Analyses for the Association of Serum PBDE Levels With All-Cause Mortality **eTable 3.** Stratified Analyses for the Association of Serum PBDE Levels With CVD Mortality **eTable 4.** Association of Serum PBDE Congeners Levels With All-Cause and Cause-Specific Mortality **eTable 5.** Association of Serum PBDE Levels With All-Cause and Cause-Specific Mortality After Excluding Participants Younger Than 40 Years Old

Supplement 2.
Data Sharing Statement
