# Supplementary material for: Exposure to Polybrominated Diphenyl Ethers and Risk of All-Cause and Cause-Specific Mortality
Source: JAMA Netw Open. 2024 Apr 1;7(4):e243127. doi: 10.1001/jamanetworkopen.2024.3127 (PMC10985557; doi:10.1001/jamanetworkopen.2024.3127)
Supplement: Supplement 1. — eFigure. Flow Chart eTable 1. Characteristics of the Study Population (n=1,100) eTable 2. Stratified Analyses for the Association of Serum PBDE Levels With All-Cause Mortality eTable 3. Stratified Analyses for the Association of Serum PBDE Levels With CVD Mortality eTable 4. Association of Serum PBDE Congeners Levels With All-Cause and Cause-Specific Mortality eTable 5. Association of Serum PBDE Levels With All-Cause and Cause-Specific Mortality After Excluding Participants Younger Than 40 Years Old [file jamanetwopen-e243127-s001.pdf]

Supplemental Online Content

Liu B, Lehmler H, Ye Z, et al. Exposure to polybrominated diphenyl ethers and risk of all-cause and cause-specific mortality. *JAMA Netw Open.* 2024;7(3):e243127. doi:10.1001/jamanetworkopen.2024.3127

eFigure 1. Flow Chart

eTable 1. Characteristics of the Study Population (n=1,100)

eTable 2. Stratified Analyses for the Association of Serum PBDE Levels With All-Cause Mortality

eTable 3. Stratified Analyses for the Association of Serum PBDE Levels With CVD Mortality

eTable 4. Association of Serum PBDE Congeners Levels With All-Cause and Cause-Specific Mortality

eTable 5. Association of Serum PBDE Levels With All-Cause and Cause-Specific Mortality After Excluding Participants Younger Than 40 Years Old

This supplemental material has been provided by the authors to give readers additional information about their work.

© 2024 Liu B et al. *JAMA Network Open.*

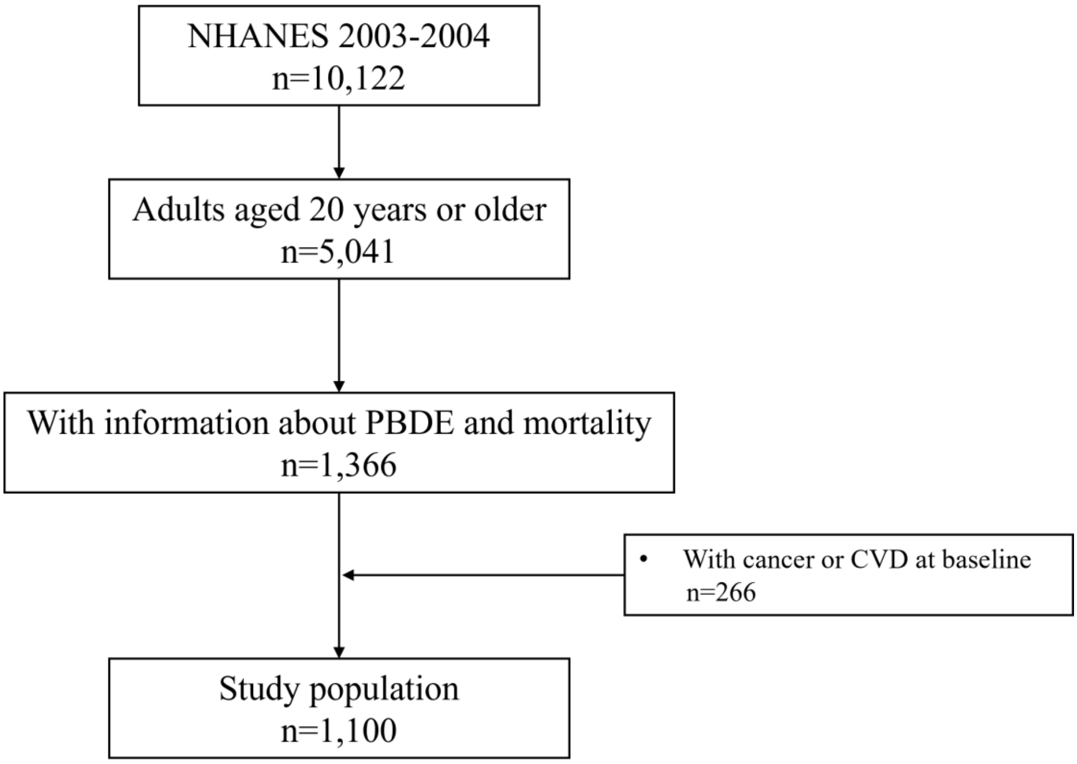

eFigure 1. Flow chart for participant inclusion

© 2024 Liu B et al. *JAMA Network Open.*

eTable 1. Characteristics of the study population (n=1,100).

Characteristic	No. of participants	Tertile 1	Tertile 2	Tertile 3	P
2,4,4'-tribromodiphenyl ether (BR28)					
Number of participants	1100	362	371	367	0·10
Age, years	1100	43·6±1·2	41·1±0·7	43·9±0·7	
Sex, %					
Male	516	48·1 (3·4)	47·8 (2·8)	48·6 (3·4)	0·98
Female	584	51·9 (3·4)	52·2 (2·8)	51·4 (3·4)	
Race/ethnicity, %					
Hispanic	293	10·5 (2·7)	14·9 (3·2)	13·3 (3·1)	
Non-Hispanic whites	544	73·2 (4·9)	68·7 (4·1)	70·5 (4·1)	0·76
Non-Hispanic black	210	10·4 (2·5)	10·6 (2·1)	10·5 (1·9)	
Other	53	5·8 (1·8)	5·8 (1·5)	5·8 (1·3)	
Education, %					
Less than high school	305	14·4 (2·5)	18·3 (2·1)	16·2 (2·0)	
High school	275	30·5 (3·0)	21·9 (4·3)	27·8 (3·5)	0·34
College or higher	520	55·1 (2·7)	59·8 (4·8)	56·0 (3·8)	
Family income to poverty ratio, %					
<1·0	207	11·4 (2·1)	16·3 (2·9)	12·1 (2·3)	
1·0-1·9	260	17·8 (2·3)	15·6 (3·3)	21·1 (2·1)	
2·0-3·9	281	26·9 (3·0)	32·3 (4·3)	27·4 (2·4)	0·45
≥4·0	286	38·1 (3·2)	29·7 (5·8)	33·6 (3·4)	
Missing	66	5·8 (1·1)	6·1 (2·1)	5·8 (1·1)	
Smoking, %					
Never smoker	580	45·9 (4·5)	52·8 (3·1)	54·6 (3·8)	
Ever smoker	243	21·9 (3·9)	23·5 (2·7)	18·4 (2·6)	0·30
Current smoker	277	32·3 (4·3)	23·8 (3·3)	26·9 (3·5)	

© 2024 Liu B et al. *JAMA Network Open.*

Alcohol drinking, %					
Non-drinker	790	72·7 (4·5)	65·1 (3·9)	70·1 (3·4)	
Moderate drinking	97	8·8 (1·6)	8·8 (2·1)	11·4 (1·7)	0·17
Heavy drinking	169	15·1 (3·4)	23·7 (2·5)	16·5 (2·7)	
Missing	44	3·4 (1·2)	2·4 (0·7)	2·0 (0·6)	
Physical activity categories*, %					
Below	518	42·7 (3·1)	36·2 (3·5)	43·8 (2·8)	
Meet	174	17·7 (2·5)	19·1 (2·8)	14·4 (2·8)	0·23
Exceed	408	39·6 (3·8)	44·7 (3·4)	41·8 (2·9)	
Total energy intake, kcal/d	1056	2315·0±72·5	2365·8±57·1	2317·8±46·1	0·53
HEI-2010 score	1056	46·0±1·2	44·7±1·0	45·4±0·9	0·33
BMI categories, %					
<25 kg/m2	349	40·5 (2·5)	30·7 (3·2)	29·5 (3·4)	
25-29·9 kg/m2	375	31·2 (2·4)	30·0 (2·8)	35·9 (3·2)	0·03
≥30 kg/m2	356	26·8 (2·5)	38·4 (3·5)	33·6 (3·1)	
Missing	20	1·5 (0·7)	1·4 (0·7)	1·1 (0·4)	
2,2',4,4'-tetrabromphenyl ether (BR47)					
Number of participants	1100	365	369	366	
Age, years	1100	44·4±1·0	42·3±0·8	41·9±1·0	0·03
Sex, %					
Male	516	48·5 (3·2)	48·5 (2·6)	47·4 (3·3)	0·95
Female	584	51·5 (3·2)	51·5 (2·6)	52·6 (3·3)	
Race/ethnicity, %					
Hispanic	293	10·7 (2·4)	14·8 (3·0)	13·0 (3·1)	
Non-Hispanic whites	544	73·2 (4·7)	69·4 (4·0)	69·9 (4·1)	0·53
Non-Hispanic black	210	10·3 (2·3)	9·2 (1·6)	12·1 (2·2)	
Other	53	5·7 (1·8)	6·6 (2·2)	5·0 (1·1)	
Education, %					

© 2024 Liu B et al. *JAMA Network Open.*

Less than high school	305	15·2 (2·3)	17·0 (2·4)	16·5 (2·3)	
High school	275	28·2 (2·5)	24·2 (3·7)	28·1 (3·4)	0·76
College or higher	520	56·6 (2·7)	58·8 (5·0)	55·4 (3·7)	
Family income to poverty ratio, %					
<1·0	207	13·1 (2·6)	12·2 (2·0)	14·6 (2·0)	
1·0-1·9	260	18·4 (2·4)	16·0 (2·3)	20·2 (1·8)	
2·0-3·9	281	26·4 (2·6)	33·6 (4·0)	26·4 (2·7)	0·40
≥4·0	286	35·6 (4·1)	33·9 (4·2)	31·8 (3·5)	
Missing	66	6·5 (1·5)	4·3 (1·6)	6·9 (1·5)	
Smoking, %					
Never smoker	580	51·2 (4·3)	48·4 (3·5)	52·5 (4·1)	
Ever smoker	243	21·7 (3·5)	24·8 (2·5)	17·1 (2·8)	0·42
Current smoker	277	27·2 (3·1)	25·8 (3·5)	30·4 (3·5)	
Alcohol drinking, %					
Non-drinker	790	70·9 (4·4)	67·4 (3·9)	69·7 (3·3)	
Moderate drinking	97	9·5 (2·0)	11·0 (1·9)	8·3 (1·5)	0·7
Heavy drinking	169	17·0 (3·2)	18·1 (2·8)	20·2 (2·7)	
Missing	44	2·5 (1·0)	3·5 (1·1)	1·8 (0·6)	
Physical activity categories*, %					
Below	518	42·2 (3·4)	37·0 (3·3)	43·7 (2·9)	
Meet	174	19·7 (3·6)	14·6 (1·3)	17·1 (2·9)	0·09
Exceed	408	38·0 (4·3)	48·5 (2·6)	39·2 (3·8)	
Total energy intake, kcal/d	1056	2398·6±78·8	2260·5±76·5	2340·1±57·3	0·18
HEI-2010 score	1056	46·4±1·1	44·2±1·1	45·5±0·9	0·11
BMI categories, %					
<25 kg/m^2	349	38·9 (3·0)	30·5 (2·6)	31·7 (3·3)	
25-29·9 kg/m^2	375	32·1 (2·7)	30·9 (3·2)	33·6 (3·0)	0·23
≥30 kg/m^2	356	28·1 (3·9)	36·8 (3·2)	33·5 (3·5)	

© 2024 Liu B et al. *JAMA Network Open.*

Missing	20	1·0 (0·6)	1·8 (0·5)	1·2 (0·4)	
2,2',4,4',5-pentabromphenyl (BR99)					
Number of participants	1100	366	368	366	
Age, years	1100	45·3±1·2	41·8±0·7	41·2±1·1	0·001
Sex, %					
Male	516	46·9 (3·8)	47·1 (3·3)	50·7 (3·5)	0·7
Female	584	53·1 (3·8)	52·9 (3·3)	49·3 (3·5)	
Race/ethnicity, %					
Hispanic	293	7·9 (1·8)	15·8 (3·9)	15·4 (3·4)	0·06
Non-Hispanic whites	544	77·5 (3·9)	67·7 (5·1)	66·5 (4·5)	
Non-Hispanic black	210	8·3 (1·7)	10·3 (2·1)	13·1 (2·2)	
Other	53	6·3 (2·0)	6·1 (1·9)	4·9 (1·3)	
Education, %					
Less than high school	305	12·2 (2·0)	20·6 (2·3)	16·5 (2·4)	0·06
High school	275	29·0 (3·0)	21·6 (3·3)	29·5 (3·6)	
College or higher	520	58·8 (3·2)	57·8 (3·7)	54·0 (3·8)	
Family income to poverty ratio, %					
<1·0	207	11·4 (2·3)	14·7 (1·9)	13·9 (1·9)	0·68
1·0-1·9	260	16·8 (2·3)	18·6 (2·4)	19·2 (2·2)	
2·0-3·9	281	30·5 (2·9)	27·4 (3·2)	28·6 (2·6)	
≥4·0	286	35·9 (3·9)	34·3 (4·1)	31·0 (3·3)	
Missing	66	5·5 (1·4)	5·0 (1·8)	7·2 (1·5)	
Smoking, %					
Never smoker	580	48·6 (4·2)	50·8 (3·0)	53·8 (3·5)	0·40
Ever smoker	243	23·9 (3·8)	23·1 (2·3)	16·6 (2·7)	
Current smoker	277	27·5 (4·2)	26·1 (2·4)	29·6 (3·5)	
Alcohol drinking, %					
Non-drinker	790	69·4 (4·0)	69·8 (4·0)	68·4 (3·2)	0·92

© 2024 Liu B et al. *JAMA Network Open.*

Moderate drinking	97	10·6 (2·7)	9·1 (1·6)	9·2 (1·5)	
Heavy drinking	169	17·2 (3·2)	18·0 (3·1)	20·2 (2·3)	
Missing	44	2·6 (0·9)	3·1 (1·1)	2·2 (0·8)	
Physical activity categories*, %					
Below	518	40·5 (3·9)	38·7 (3·3)	43·5 (3·4)	
Meet	174	18·7 (3·3)	17·3 (1·4)	15·1 (2·1)	0·62
Exceed	408	40·7 (4·3)	44·0 (3·2)	41·4 (3·7)	
Total energy intake, kcal/d	1056	2348·5±82·7	2355·4±83·8	2292·8±46·5	0·95
HEI-2010 score	1056	46·8±1·0	43·9±1·0	45·3±0·9	0·02
BMI categories, %					
<25 kg/m^2	349	34·2 (2·9)	32·6 (2·1)	34·3 (2·8)	
25-29·9 kg/m^2	375	31·9 (2·9)	32·2 (2·9)	32·4 (2·6)	0·99
≥30 kg/m^2	356	32·5 (3·9)	33·9 (3·1)	32·0 (3·2)	
Missing	20	1·4 (0·8)	1·3 (0·7)	1·2 (0·4)	
2,2',4,4',6-pentabromdphenyl (BR100)					
Number of participants	1100	367	366	367	
Age, years	1100	45·4±0·9	41·2±0·6	41·8±0·9	0·0001
Sex, %					
Male	516	47·2 (2·7)	45·6 (2·8)	51·9 (2·6)	0·22
Female	584	52·8 (2·7)	54·4 (2·8)	48·1 (2·6)	
Race/ethnicity, %					
Hispanic	293	9·8 (2·2)	15·9 (3·6)	13·1 (3·3)	
Non-Hispanic whites	544	75·3 (4·4)	67·1 (4·5)	70·0 (4·5)	0·12
Non-Hispanic black	210	8·9 (2·4)	10·1 (1·5)	12·7 (2·3)	
Other	53	6·1 (1·7)	6·9 (1·6)	4·3 (1·1)	
Education, %					
Less than high school	305	13·7 (2·2)	17·1 (1·5)	18·1 (2·5)	0·17
High school	275	29·9 (3·3)	22·2 (3·5)	28·1 (3·1)	

© 2024 Liu B et al. *JAMA Network Open.*

College or higher	520	56·4 (3·2)	60·6 (3·8)	53·7 (3·0)	
Family income to poverty ratio, %					
<1·0	207	12·0 (2·2)	13·1 (2·3)	14·8 (2·0)	
1·0-1·9	260	16·4 (1·8)	18·9 (2·8)	19·3 (2·0)	
2·0-3·9	281	28·1 (2·7)	30·7 (4·2)	27·7 (2·3)	0·55
≥4·0	286	36·2 (3·2)	33·5 (5·2)	31·5 (2·6)	
Missing	66	7·2 (1·5)	3·8 (1·3)	6·6 (1·8)	
Smoking, %					
Never smoker	580	52·9 (3·8)	46·5 (2·6)	53·6 (3·1)	
Ever smoker	243	21·7 (3·1)	25·2 (3·1)	16·8 (2·5)	0·12
Current smoker	277	25·4 (3·7)	28·3 (2·6)	29·6 (2·6)	
Alcohol drinking, %					
Non-drinker	790	67·8 (4·7)	72·5 (3·6)	67·7 (2·3)	
Moderate drinking	97	10·4 (2·4)	6·7 (1·3)	11·9 (1·7)	0·38
Heavy drinking	169	19·3 (3·7)	17·3 (2·8)	18·6 (2·8)	
Missing	44	2·5 (0·7)	3·6 (1·0)	1·8 (0·8)	
Physical activity categories*, %					
Below	518	40·5 (3·7)	40·4 (3·2)	41·8 (2·6)	
Meet	174	18·5 (2·7)	17·1 (2·5)	15·6 (2·2)	0·84
Exceed	408	41·0 (4·3)	42·4 (2·3)	42·7 (3·6)	
Total energy intake, kcal/d	1056	2378·1±71·7	2237·1±68·0	2382·4±49·7	0·053
HEI-2010 score	1056	46·8±1·1	44·3±1·3	45·0±0·8	0·09
BMI categories, %					
<25 kg/m^2	349	35·1 (3·2)	35·3 (2·1)	30·5 (3·5)	
25-29·9 kg/m^2	375	33·8 (2·6)	27·1 (2·9)	35·7 (3·7)	0·41
≥30 kg/m^2	356	29·9 (4·4)	35·8 (3·3)	32·9 (3·3)	
Missing	20	1·3 (0·8)	1·8 (0·6)	0·9 (0·4)	

© 2024 Liu B et al. *JAMA Network Open.*

2,2',4,4',5,5'-hexabromphenyl (BR153)					
Number of participants	1100	361	374	365	
Age, years	1100	46·1±0·8	41·5±0·7	41·1±1·0	0·0002
Sex, %					
Male	516	39·8 (2·9)	48·7 (3·2)	55·7 (3·3)	0·01
Female	584	60·2 (2·9)	51·3 (3·2)	44·3 (3·3)	
Race/ethnicity, %					
Hispanic	293	11·4 (2·3)	17·5 (4·6)	9·5 (2·6)	
Non-Hispanic whites	544	71·2 (4·7)	67·4 (5·2)	74·1 (3·9)	0·25
Non-Hispanic black	210	11·0 (2·8)	9·2 (1·6)	11·3 (1·8)	
Other	53	6·4 (1·7)	5·8 (1·6)	5·2 (1·7)	
Education, %					
Less than high school	305	15·4 (2·4)	16·0 (1·6)	17·3 (2·3)	
High school	275	25·5 (2·6)	25·0 (4·1)	29·8 (4·3)	0·61
College or higher	520	59·0 (2·9)	59·0 (3·6)	52·8 (4·2)	
Family income to poverty ratio, %					
<1·0	207	11·4 (1·8)	14·9 (2·9)	13·4 (2·3)	
1·0-1·9	260	14·3 (1·6)	17·9 (2·4)	22·1 (3·4)	
2·0-3·9	281	30·4 (3·5)	29·2 (3·4)	27·1 (2·1)	0·09
≥4·0	286	35·0 (3·5)	35·3 (4·8)	31·2 (3·4)	
Missing	66	8·9 (1·9)	2·7 (0·8)	6·2 (1·3)	
Smoking, %					
Never smoker	580	51·5 (3·3)	54·8 (3·1)	46·6 (2·9)	
Ever smoker	243	25·5 (2·9)	20·1 (3·4)	18·5 (2·0)	0·005
Current smoker	277	23·0 (2·9)	25·2 (2·6)	34·9 (3·0)	
Alcohol drinking, %					
Non-drinker	790	68·7 (4·9)	71·0 (3·9)	68·2 (2·5)	0·70
Moderate drinking	97	10·4 (2·3)	7·6 (1·6)	11·0 (1·8)	

© 2024 Liu B et al. *JAMA Network Open.*

Heavy drinking	169	18·0 (3·7)	18·1 (2·9)	19·2 (2·5)	
Missing	44	2·9 (1·1)	3·4 (1·3)	1·5 (0·8)	
Physical activity categories*, %					
Below	518	39·8 (3·7)	42·1 (5·1)	40·7 (2·8)	
Meet	174	21·2 (3·3)	15·4 (2·0)	15·0 (2·6)	0·42
Exceed	408	39·0 (4·0)	42·5 (3·8)	44·4 (3·7)	
Total energy intake, kcal/d	1056	2248·8±66·3	2338·7±77·2	2407·2±76·4	0·28
HEI-2010 score	1056	45·4±1·0	46·2±1·0	44·5±0·8	0·48
BMI categories, %					
<25 kg/m^2	349	28·6 (2·8)	33·1 (3·4)	39·2 (3·3)	
25-29·9 kg/m^2	375	31·1 (2·6)	32·2 (4·3)	33·2 (3·5)	0·25
≥30 kg/m^2	356	38·8 (3·8)	33·5 (3·9)	26·3 (3·5)	
Missing	20	1·5 (0·9)	1·2 (0·4)	1·3 (0·6)	
2,2',4,4',5,5'-hexbrombiphenyl (BB1L)					
Number of participants	1099	372	360	367	
Age, years	1099	35·1±0·8	44·9±1·1	47·6±0·7	<0·0001
Sex, %					
Male	516	37·9 (3·6)	48·1 (3·6)	57·4 (2·5)	0·0004
Female	584	62·1 (3·6)	51·9 (3·6)	42·6 (2·5)	
Race/ethnicity, %					
Hispanic	293	23·0 (3·1)	9·8 (2·9)	7·0 (2·5)	
Non-Hispanic whites	543	56·4 (4·2)	79·0 (3·9)	75·2 (4·0)	<0·0001
Non-Hispanic black	210	11·1 (1·6)	7·5 (1·7)	13·2 (2·5)	
Other	53	9·6 (2·3)	3·8 (1·3)	4·6 (1·5)	
Education, %					
Less than high school	305	19·5 (2·4)	14·0 (2·2)	15·8 (2·3)	
High school	275	25·1 (4·0)	25·9 (3·1)	29·2 (3·0)	0·39
College or higher	520	55·4 (3·6)	60·1 (2·7)	55·0 (3·0)	

© 2024 Liu B et al. *JAMA Network Open.*

Family income to poverty ratio, %					
<1·0	207	18·5 (2·4)	8·4 (1·5)	13·8 (2·7)	
1·0-1·9	260	19·4 (2·7)	21·1 (3·0)	13·9 (2·1)	
2·0-3·9	281	28·1 (2·8)	28·9 (2·5)	29·6 (2·7)	0·03
≥4·0	286	27·0 (3·0)	36·5 (4·0)	37·1 (4·6)	
Missing	66	7·0 (1·8)	5·1 (0·9)	5·7 (1·9)	
Smoking, %					
Never smoker	580	56·9 (2·3)	48·9 (3·0)	47·9 (3·2)	
Ever smoker	243	16·9 (1·9)	24·4 (3·5)	21·9 (2·7)	0·08
Current smoker	277	26·2 (3·1)	26·7 (2·5)	30·2 (3·1)	
Alcohol drinking, %					
Non-drinker	790	69·1 (4·0)	72·7 (3·3)	65·9 (3·4)	
Moderate drinking	97	9·6 (1·8)	11·0 (2·3)	8·2 (1·7)	
Heavy drinking	169	17·8 (2·9)	14·3 (2·8)	23·4 (3·2)	0·20
Missing	44	3·2 (0·9)	2·0 (0·7)	2·5 (1·1)	
Physical activity categories*, %					
Below	518	46·3 (2·4)	39·5 (3·6)	37·5 (3·0)	
Meet	174	17·1 (2·6)	17·3 (2·9)	17·0 (2·9)	0·27
Exceed	408	36·6 (3·4)	43·2 (3·6)	45·5 (3·4)	
Total energy intake, kcal/d	1056	2386·0±72·0	2287·4±73·4	2334·3±46·2	0·30
HEI-2010 score	1056	45·3±1·2	45·1±1·3	45·8±1·0	0·87
BMI categories, %					
<25 kg/m2	349	33·3 (2·4)	28·4 (2·3)	39·8 (4·5)	
25-29·9 kg/m2	375	25·8 (2·5)	35·7 (3·2)	34·2 (2·3)	
≥30 kg/m2	356	39·8 (2·3)	34·2 (3·9)	25·0 (4·6)	0·03
Missing	20	1·2 (0·7)	1·7 (0·7)	1·0 (0·7)	

Abbreviations: BMI, body mass index; HEI, healthy eating index; MET, metabolic equivalent of task.

Values were weighted mean ± standard error (SE) for continuous variables and weighted percentages (SE) for categorical variables, except the number of participants.

© 2024 Liu B et al. *JAMA Network Open.*

* Physical activity for each participant was categorized as follows: (i) below, 150 min/week moderate- to vigorous-intensity activity; (ii) meet, 150-300 min/week moderate- to vigorous-intensity activity; or (iii) exceed, 300 min/week moderate- to vigorous-intensity activity.

© 2024 Liu B et al. *JAMA Network Open.*

eTable 2. Stratified analyses for the association of serum PBDE levels with all-cause mortality.

		Tertile 1	Tertile 2	Tertile 3	P for interaction
Age	<60 years	21/3961	11/4531	14/4335	0·54
		1 (ref)	0·36 (0·18-0·70)	0·38 (0·18-0·80)	
	≥60 years	53/1336	44/981	56/1019	
		1 (ref)	1·18 (0·62-2·27)	2·10 (1·37-3·22)	
Sex	Male	31/2380	30/2371	43/2717	0·85
		1 (ref)	1·11 (0·57-2·16)	1·29 (0·70-2·37)	
	Female	43/2916	25/3141	27/2637	
		1 (ref)	0·50 (0·26-0·99)	1·22 (0·60-2·48)	
Race/ethnicity	Whites	44/2842	21/2522	38/2587	0·34
		1 (ref)	0·60 (0·37-0·95)	1·29 (0·83-2·00)	
	Non-whites	30/2454	34/2990	32/2767	
		1 (ref)	1·33 (0·60-2·98)	1·16 (0·48-2·84)	
Diet quality[#]	Lower	30/2705	31/2379	35/2647	0·73
		1 (ref)	0·98 (0·52-1·84)	1·54 (0·74-3·20)	
	Higher	44/2591	24/3133	35/2706	
		1 (ref)	1·04 (0·52-2·08)	1·76 (0·997-3·10)	
Physical activity[*]	Lower	54/2467	39/2316	46/2610	0·82
		1 (ref)	0·79 (0·51-1·22)	1·55 (0·87-2·78)	
	Higher	20/2830	16/3196	24/2744	
		1 (ref)	0·91 (0·32-2·61)	1·24 (0·55-2·79)	
Obesity	BMI < 30	52/3546	38/3470	51/3549	0·41
		1 (ref)	0·86 (0·52-1·41)	1·63 (0·91-2·93)	
	BMI ≥ 30	21/1686	16/1921	16/1723	
		1 (ref)	0·65 (0·25-1·64)	0·78 (0·27-2·21)	

[#] Lower or higher diet quality was defined as the healthy eating index (HEI) < the median score or ≥ the median score, respectively.

[*] Lower or higher physical activity level was defined as below or meeting the physical activity guidelines, respectively.

© 2024 Liu B et al. *JAMA Network Open.*

Adjustment for age, sex, race/ethnicity, education, family income status, smoking, alcohol drinking, physical activity, total energy intake, HEI2010 score, and BMI.

© 2024 Liu B et al. *JAMA Network Open.*

eTable 3. Stratified analyses for the association of serum PBDE levels with CVD mortality.

		Tertile 1	Tertile 2	Tertile 3	P for interaction
Age	<60 years	6/3961	2/4531	2/4335	0·09
		1 (ref)	0·03 (0·002-0·44)	0·003 (0·00-9·28)	
	≥60 years	19/1336	19/981	16/1019	
		1 (ref)	1·13 (0·41-3·10)	1·18 (0·49-2·81)	
Sex	Male	10/2380	6/2371	14/2717	0·44
		1 (ref)	0·31 (0·06-1·78)	1·15 (0·36-3·64)	
	Female	15/2916	15/3141	4/2637	
		1 (ref)	1·39 (0·50-3·85)	0·50 (0·11 -2·34)	
Race/ethnicity	Whites	12/2842	7/2522	10/2587	0·50
		1 (ref)	0·39 (0·12-1·35)	0·52 (0·20-1·34)	
	Non-whites	13/2454	14/2990	8/2767	
		1 (ref)	1·69 (0·73-3·91)	1·47 (0·32-6·71)	
Diet quality[#]	Lower	10/2705	11/2379	8/2647	0·85
		1 (ref)	1·69 (0·53-5·38)	1·09 (0·25-4·79)	
	Higher	15/2591	10/3133	10/2706	
		1 (ref)	0·48 (0·08-3·05)	0·71 (0·23-2·22)	
Physical activity[*]	Lower	22/2830	18/3196	15/2744	0·67
		1 (ref)	0·74 (0·26-2·12)	0·84 (0·32-2·23)	
	Higher	3/2467	3/2316	3/2610	
		1 (ref)	0·01 (0·00-19803·2)	1·98 (0·35-11·11)	
Obesity	BMI < 30	13/3546	15/3470	13/3549	0·02
		1 (ref)	1·08 (0·25-4·66)	1·77 (0·59-5·28)	
	BMI ≥ 30	11/1686	6/1921	4/1723	
		1 (ref)	0·12 (0·02-0·86)	0·05 (0·003-1·03)	

[#] Lower or higher diet quality was defined as the healthy eating index (HEI) < the median score or ≥ the median score, respectively.

[*] Lower or higher physical activity level was defined as below or meeting the physical activity guidelines, respectively.

© 2024 Liu B et al. *JAMA Network Open.*

Adjustment for age, sex, race/ethnicity, education, family income status, smoking, alcohol drinking, physical activity, total energy intake, HEI2010 score, and BMI.

© 2024 Liu B et al. *JAMA Network Open.*

46 **eTable 4. Association of serum PBDE congeners levels with all-cause and cause-specific mortality**

2,4,4'-tribromodiphenyl ether (BR28)	Tertile 1	Tertile 2	Tertile 3	OR per unit	P for trend
Median PBDE level, ng/mL	0.4	1.1	3.0		
**All-cause mortality**					
Deaths/person-years	70/5238	59/5562	70/5362		
HR (95% CI)					
Model 1	1 (reference)	0.96 (0.65-1.43)	0.93 (0.61-1.41)	0.96 (0.78-1.19)	0.71
Model 2	1 (reference)	0.96 (0.71-1.30)	0.89 (0.61-1.29)	0.94 (0.79-1.13)	0.51
Model 3	1 (reference)	1.00 (0.74-1.33)	0.91 (0.64-1.30)	0.96 (0.81-1.13)	0.58
**CVD mortality**					
Deaths/person-years	25/5238	19/5562	20/5362		
HR (95% CI)					
Model 1	1 (reference)	0.61 (0.25-1.49)	0.60 (0.25-1.47)	0.76 (0.47-1.25)	0.26
Model 2	1 (reference)	0.66 (0.30-1.43)	0.57 (0.23-1.37)	0.75 (0.47-1.18)	0.19
Model 3	1 (reference)	0.68 (0.31-1.52)	0.57 (0.24-1.32)	0.75 (0.49-1.15)	0.17
**Cancer mortality**					
Deaths/person-years	15/5238	13/5562	24/5362		
HR (95% CI)					
Model 1	1 (reference)	1.21 (0.61-2.42)	1.59 (0.72-3.51)	1.26 (0.85-1.88)	0.23
Model 2	1 (reference)	1.27 (0.63-2.58)	1.86 (0.84-4.11)	1.37 (0.91-2.06)	0.13
Model 3	1 (reference)	1.18 (0.53-2.63)	1.80 (0.89-3.63)	1.25 (0.93-1.96)	0.11
**2,2',4,4'-tetrabromphenyl ether (BR47)**	**Tertile 1**	**Tertile 2**	**Tertile 3**		
Median PBDE level, ng/mL	7.2	19.4	56.2		
**All-cause mortality**					
Deaths/person-years	72/5289	58/5504	69/5369		
HR (95% CI)					
Model 1	1 (reference)	0.96 (0.68-1.33)	1.21 (0.80-1.83)	1.10 (0.89-1.36)	0.37
Model 2	1 (reference)	0.98 (0.67-1.43)	1.16 (0.76-1.75)	1.07 (0.87-1.32)	0.49
Model 3	1 (reference)	1.02 (0.73-1.43)	1.19 (0.81-1.75)	1.09 (0.99-1.32)	0.36
**CVD mortality**					
Deaths/person-years	26/5289	19/5504	19/5369		
HR (95% CI)					
Model 1	1 (reference)	0.60 (0.27-1.34)	0.86 (0.36-2.07)	0.91 (0.55-1.48)	0.68
Model 2	1 (reference)	0.65 (0.31-1.38)	0.95 (0.37-2.46)	0.95 (0.57-1.58)	0.83
Model 3	1 (reference)	0.64 (0.31-1.30)	0.96 (0.42-2.20)	0.95 (0.61-1.48)	0.81

© 2024 Liu B et al. *JAMA Network Open.*

**Cancer mortality**					
Deaths/person-years	10/5289	18/5504	24/5369		
HR (95% CI)					
Model 1	1 (reference)	2.00 (0.95-4.20)	**2.78 (1.22-6.32)**	**1.60 (1.11-2.31)**	**0.02**
Model 2	1 (reference)	2.01 (0.97-4.16)	**3.06 (1.17-8.04)**	**1.69 (1.06-2.68)**	**0.03**
Model 3	1 (reference)	**2.23 (1.01-4.94)**	**3.21 (1.29-7.96)**	**1.71 (1.12-2.60)**	**0.02**
**2,2',4,4',5-pentabromphenyl (BR99)**	**Tertile 1**	**Tertile 2**	**Tertile 3**		
Median PBDE level, ng/mL	1.8	4.0	12.6		
**All-cause mortality**					
Deaths/person-years	77/5283	59/5474	63/5405		
HR (95% CI)					
Model 1	1 (reference)	0.83 (0.54-1.29)	1.16 (0.76-1.76)	1.08 (0.86-1.36	0.49
Model 2	1 (reference)	0.79 (0.49-1.27)	1.10 (0.70-1.75)	1.05 (0.82-1.36)	0.66
Model 3	1 (reference)	0.79 (0.50-1.26)	1.11 (0.72-1.72)	1.06 (0.83-1.34)	0.62
**CVD mortality**					
Deaths/person-years	26/5283	22/5474	16/5405		
HR (95% CI)					
Model 1	1 (reference)	0.85 (0.39-1.85)	0.84 (0.33-2.18)	0.91 (0.55-1.51)	0.70
Model 2	1 (reference)	0.93 (0.49-1.76)	0.93 (0.35-2.44)	0.96 (0.58-1.58)	0.86
Model 3	1 (reference)	1.01 (0.53-1.93)	1.00 (0.40-2.49)	1.00 (0.63-1.59)	1.00
**Cancer mortality**					
Deaths/person-years	16/5283	15/5474	21/5405		
HR (95% CI)					
Model 1	1 (reference)	0.91 (0.48-1.76)	1.66 (0.79-3.48)	1.32 (0.87-2.02)	0.17
Model 2	1 (reference)	0.92 (0.51-1.66)	1.77 (0.70-4.46)	1.37 (0.81-2.32)	0.22
Model 3	1 (reference)	0.95 (0.54-1.67)	1.71 (0.77-3.81)	1.34 (0.65-2.10)	0.18
**2,2',4,4',6-pentabromdphenyl (BR100)**	**Tertile 1**	**Tertile 2**	**Tertile 3**		
Median PBDE level, ng/mL	1.3	3.4	11.3		
**All-cause mortality**					
Deaths/person-years	72/5309	52/5521	75/5333		
HR (95% CI)					
Model 1	1 (reference)	1.00 (0.64-1.58)	**1.46 (1.02-2.09)**	**1.20 (1.01-1.42)**	**0.04**
Model 2	1 (reference)	0.98 (0.57-1.67)	**1.57 (1.06-2.32)**	**1.24 (1.02-1.50)**	**0.03**
Model 3	1 (reference)	0.98 (0.58-1.65)	**1.60 (1.10-2.31)**	**1.25 (1.04-1.50)**	**0.02**

© 2024 Liu B et al. *JAMA Network Open.*

**CVD mortality**					
Deaths/person-years	26/5309	16/5521	22/5333		
HR (95% CI)					
Model 1	1 (reference)	0.71 (0.25-2.08)	0.95 (0.44-2.04)	0.97 (0.66-1.43)	0.86
Model 2	1 (reference)	0.71 (0.23-2.27)	1.13 (0.54-2.33)	1.04 (0.70-1.52)	0.85
Model 3	1 (reference)	0.73 (0.25-2.15)	1.11 (0.55-2.23)	1.03 (0.72-1.48)	0.86
**Cancer mortality**					
Deaths/person-years	9/5309	19/5521	24/5333		
HR (95% CI)					
Model 1	1 (reference)	2.16 (0.95-4.92)	**2.98 (1.17-7.61)**	**1.60 (1.10-2.32)**	**0.02**
Model 2	1 (reference)	2.23 (0.80-6.20)	**3.83 (1.06-13.87)**	**1.81 (1.05-3.13)**	**0.03**
Model 3	1 (reference)	2.14 (0.77-5.95)	**3.81 (1.16-12.49)**	**1.82 (1.09-3.02)**	**0.03**
**2,2',4,4',5,5'-hexabromphenyl (BR153)**	**Tertile 1**	**Tertile 2**	**Tertile 3**		
Median PBDE level, ng/mL	1.8	4.5	16.5		
**All-cause mortality**					
Deaths/person-years	67/5288	59/5555	73/5319		
HR (95% CI)					
Model 1	1 (reference)	1.33 (0.81-2.18)	**1.82 (1.21-2.75)**	**1.31 (1.09-1.57)**	**0.006**
Model 2	1 (reference)	1.32 (0.78-2.23)	**1.81 (1.19-2.75)**	**1.31 (1.09-1.56)**	**0.006**
Model 3	1 (reference)	1.31 (0.81-2.12)	**1.78 (1.16-2.73)**	**1.30 (1.08-1.56)**	**0.009**
**CVD mortality**					
Deaths/person-years	26/5288	19/5555	19/5319		
HR (95% CI)					
Model 1	1 (reference)	1.05 (0.50-2.22)	0.80 (0.34-1.88)	0.91 (0.63-1.32)	0.59
Model 2	1 (reference)	0.95 (0.45-2.04)	0.76 (0.32-1.85)	0.89 (0.60-1.32)	0.52
Model 3	1 (reference)	1.03 (0.47-2.26)	0.72 (0.31-1.65)	0.86 (0.60-1.24)	0.40
**Cancer mortality**					
Deaths/person-years	11/5288	19/5555	22/5319		
HR (95% CI)					
Model 1	1 (reference)	2.27 (0.86-5.96)	**3.47 (1.27-9.48)**	**1.67 (1.10-2.53)**	**0.02**
Model 2	1 (reference)	2.54 (0.86-7.50)	**4.35 (1.36-13.88)**	**1.84 (1.17-2.91)**	**0.01**
Model 3	1 (reference)	2.39 (0.86-6.64)	**4.12 (1.37-12.42)**	**1.81 (1.18-2.78)**	**0.01**

Model 1: adjustment for age, sex, and race/ethnicity.

Model 2: model 1 + education, family income status, smoking, alcohol drinking, physical activity, total energy intake, HEI-2010 score, and BMI.

© 2024 Liu B et al. *JAMA Network Open.*

eTable 5. Association of serum PBDE levels with all-cause and cause-specific mortality after excluding participants younger than 40 years old

	Tertile 1	Tertile 2	Tertile 3	OR per unit	P for trend
Median PBDE level, ng/mL	14.85	33.5	102.7		
All-cause mortality					
Deaths/person-years	62/2855	56/2992	71/2796		
HR (95% CI)					
Model 1	1 (reference)	1.12 (0.71-1.76)	1.37 (0.78-2.40)	1.18 (0.89-1.56)	0.24
Model 2	1 (reference)	1.09 (0.63-1.88)	1.31 (0.77-2.23)	1.15 (0.88-1.51)	0.29
Model 3	1 (reference)	1.09 (0.64-1.86)	1.34 (0.81-2.21)	1.16 (0.90-1.51)	0.23
CVD mortality					
Deaths/person-years	23/2855	20/2992	19/2796		
HR (95% CI)					
Model 1	1 (reference)	0.77 (0.30-1.98)	0.79 (0.27-2.32)	0.88 (0.49-1.58)	0.65
Model 2	1 (reference)	0.66 (0.24-1.82)	0.72 (0.22-2.36)	0.83 (0.42-1.63)	0.57
Model 3	1 (reference)	0.68 (0.26-1.76)	0.76 (0.26-2.27)	0.86 (0.46-1.59)	0.60
Cancer mortality					
Deaths/person-years	8/2855	18/2992	25/2796		
HR (95% CI)					
Model 1	1 (reference)	2.25 (1.05-5.73)	3.30 (1.43-7.60)	1.71 (1.22-2.40)	0.004
Model 2	1 (reference)	2.43 (0.98-6.05)	4.07 (1.49-11.13)	1.95 (1.21-3.12)	0.009
Model 3	1 (reference)	2.32 (0.99-5.45)	4.01 (1.61-9.98)	1.94 (1.27-2.99)	0.005

Model 1: adjustment for age, sex, and race/ethnicity.

Model 2: model 1 + education, family income status, smoking, alcohol drinking, physical activity, total energy intake, and HEI-2010 score.

Model 3: Model 2 + BMI.
