# Supplementary material for: Exposure to Polybrominated Diphenyl Ethers and Risk of All-Cause and Cause-Specific Mortality
Source: JAMA Netw Open. 2024 Apr 1;7(4):e243127. doi: 10.1001/jamanetworkopen.2024.3127 (PMC10985557; doi:10.1001/jamanetworkopen.2024.3127)
Supplement: Supplement 2. — Data Sharing Statement [file jamanetwopen-e243127-s002.pdf]

Data Sharing Statement

Liu. Exposure to Polybrominated Diphenyl Ethers and Risk of All-Cause and Cause-Specific Mortality. *JAMA Netw Open*. Published March 20, 2024.
doi:10.1001/jamanetworkopen.2024.3127

Data
Data available: No
